# Treatment Adherence and Persistence of Anti-Fibrotic Drugs in Real Life in Greece

**DOI:** 10.3390/arm94010006

**Published:** 2026-01-08

**Authors:** Georgia Kourlaba, Stylianos Ravanidis, Garyfallia Stefanou, Konstantinos Mathioudakis, Anastasios Tsolakidis, Dimitrios Zografopoulos

**Affiliations:** 1 Department of Nursing, University of the Peloponnese, Erythrou Stavrou (Terma), 22100 Tripoli, Greece; s.ravanidis@econcare.gr (S.R.); g.stefanou@econcare.gr (G.S.); 2 Department of Nursing, National and Kapodistrian University of Athens (NKUA), Papadiamantopoulou 123, 11527 Athens, Greece; 3 IDIKA SA—e-Government Center for Social Security Services, Lykoyrgou 10, 10551 Athens, Greece; mathioudakis@idika.gr (K.M.);; 4 Department of Archival, Library and Information Studies, University of West Attica, 12243 Egaleo, Greece; 5 Ministry of Health, Aristotelous 17, 10433 Athens, Greece; dzografopoulos@hotmail.com

**Keywords:** idiopathic pulmonary fibrosis, treatment persistence, treatment adherence, interstitial lung diseases, anti-fibrotic drugs, real-world evidence, Greece

## Abstract

**Highlights:**

**What are the main findings?**

- The overall median treatment persistence was 40.2 months with 79%, and 63% of patients remaining persistent at 12 and 24 months, respectively. Women, younger patients and patients with progressive pulmonary fibrosis diagnosis had higher treatment persistence rates compared to their counterparts.
- Adherence levels remained high across the follow-up period (90%), while older age was associated with improved adherence rates.

**What are the implications of the main findings?**

- Improvement in the clinical management of inhibitory factors, such as adverse events, may improve the persistence rates for men, which represent the majority of patients with interstitial pulmonary fibrosis (IPF) diagnosis.
- Clinical and demographic factors of each patient should be considered before the launch of anti-fibrotic therapy in order to secure the maximal therapeutic benefits.

**Abstract:**

Background: Nintedanib and pirfenidone are two anti-fibrotic agents for diseases within the interstitial lung diseases (ILDs) spectrum. Here, we provide a comprehensive analysis regarding treatment persistence and adherence rates for the Greek territory. Methods: This was a retrospective cohort study of patients initiating anti-fibrotic treatment during the period 2019–2023, utilizing data extracted from the National Electronic Prescription Database. Treatment persistence was defined as the duration from the date of the first prescription to the end of follow-up, death, or switching to another agent. Adherence was estimated based on the Medication Possession Ratio (MPR) metric. Results: Overall, 2112 patients were analyzed. The majority were naive, male patients with a diagnosis of idiopathic pulmonary fibrosis (IPF). The overall median treatment persistence was 40.2 months (95% CI: 35.5–44.6). Women and treatment-naive patients demonstrated longer median treatment persistence compared to their counterparts, while older patients demonstrated the lowest median persistence rates. Adherence levels remained high across the follow-up period (90%). Diagnosis of IPF and gastrointestinal comorbidities were associated with a higher risk of discontinuation. Conclusions: We have generated novel data concerning the factors that affect patients’ outcomes under anti-fibrotic therapy. These findings may provide helpful insights for the therapeutic management of ILDs.

## 1. Introduction

The group of interstitial lung diseases (ILDs) comprises approximately 200 diseases that are characterized by respiratory symptoms including progressive dyspnoea, cough, and impaired lung function due to persistent inflammation and subsequent scarring of lung tissue [1]. The therapeutic landscape of ILDs has evolved significantly since the advent of anti-fibrotic therapies, namely nintedanib and pirfenidone [2]. Both are licensed for the treatment of idiopathic pulmonary fibrosis (IPF) since they have been proven efficient in reducing the rate of decline in forced vital capacity (FVC) with a similar rate of adverse events compared to each placebo arm [3,4]. Moreover, the efficacy of nintedanib is evident for the treatment of systemic sclerosis-associated (SSc)-ILDs [5] and progressive fibrosing ILDs [6]. Additionally, a recent meta-analysis has provided initial data suggesting that both anti-fibrotic drugs may be beneficial for patients suffering from a series of ILDs other than IPF, although these results warrant further investigation [2].

Given that several factors such as adverse events, disease progression and performance status may lead to treatment discontinuation or even switch between the two anti-fibrotic agents [7,8], real-world evidence studies reporting on the treatment adherence and persistence may provide essential data considering the existence of long-term benefits of anti-fibrotic therapies [9]. However, apart from IPF, this issue is not covered by the existent literature. Particularly for IPF, some studies have employed national and hospital-based registries to evaluate the treatment persistence of both anti-fibrotics [9,10,11,12]. Reported persistence rates for the first year of treatment lie between 70% and 80% [9,10], while in the second year these rates seem to drop significantly [9].

Real-world evidence from the longitudinal utilization of both anti-fibrotic agents in patients from Greece corroborate their effectiveness in terms of survival and lung function as well as their safety. Particularly, nintedanib has reduced the rate of lung function decline in patients with connective tissue disease (CTD)–ILD [13] and IPF [14,15], also accompanied by high survival and low discontinuation rates. Similarly, administration of pirfenidone has improved functional lung capacity in patients with IPF [16]. However, data concerning the treatment persistence rates of both agents for any of the indications across the ILD spectrum are lacking. Therefore, the evaluation of these rates in real-world settings will provide additional insights of great value towards the improvement of the therapeutic management of ILDs in the country.

In this study, the largest national database of electronic prescriptions has been employed to assess treatment persistence and adherence patterns of anti-fibrotic agents in Greece, as well as to identify demographic and clinical factors that are associated with the observed patterns.

## 2. Materials and Methods

### 2.1. Study Design

This was a retrospective, observational cohort study of patients receiving anti-fibrotic therapies (pirfenidone: any available brand name; nintedanib: under Ofev^®^ [Boehringer Ingelheim, Ingelheim am Rhein, Germany] brand name) in Greece. The study utilized secondary prescription data extracted from the National Electronic Prescription Database (IDIKA).

### 2.2. Study Population

Patients were included in the study population if they met all the following criteria: (1) they had initiated anti-fibrotic treatment between 1 January 2019, and 31 December 2023, either as naive or experienced, and (2) were adults at the time of their first executed prescription. Given that a treatment break could exceed six months, only patients who received their first prescription after 1 January 2020 were categorized as naive, while patients who underwent treatment during 2019 and switched to a different agent during the data-capturing period were categorized as experienced patients. Specifically, for the experienced patients, the prescriptions received before the switch were excluded from the analysis.

### 2.3. Data Collection

All prescriptions executed between 1 January 2019 and 31 December 2023 were extracted in June 2024 from the IDIKA database. The extracted variables included the following: patient demographics (sex, birth year), prescription details [issue date, date of availability for purchase, execution date, full medicine commercial name including information about the package, quantity of medicine units and drug content, the quantity of packages prescribed and executed, the duration of the medicine administration (prescription supply), International Classification of Diseases (ICD-10) codes], and additional information such as the date of death, the geographic region of the prescribing physician, and the therapeutic protocols in which the patients were registered before and during study period (2019–2023).

### 2.4. Diagnosis and Comorbidities Definition

All patients were classified into one of the following diagnoses based on the ICD-10 codes of the prescriptions received during the data capturing period: IPF, unclassifiable pulmonary fibrosis (PF), rheumatoid arthritis (RA)-associated ILD, SSc-ILD, systemic lupus erythematosus (SLE)-associated ILD, Sjogren syndrome, dermatomyositis, polymyositis, myositis, and other PF-ILDs. The detailed classification criteria are provided in Appendix A and Table S1. Patients with diagnoses other than IPF were collectively classified as progressive pulmonary fibrosis (PPF).

Comorbidities were identified using the Therapeutic Health Protocols, in which patients were registered. These were classified into various medical conditions according to the Greek Ministry of Health (full list in https://www.moh.gov.gr/, accessed on 31 June 2025) (Table S2).

### 2.5. Estimation of Treatment Persistence and Adherence

Treatment persistence was defined as the duration from the date of the first prescription of anti-fibrotic therapy to the earliest date of the following: end of follow-up, death, or switching to another anti-fibrotic agent. Notably, this definition did not consider temporary gaps in treatment as discontinuation; patients were considered persistent regardless of treatment interruptions or delays in prescription refills. Treatment gaps were instead reflected in the calculation of medication adherence, which captured the consistency of daily drug availability throughout the observation period. As sensitivity analysis, treatment persistence was additionally defined using a gap-based approach. Under this definition, treatment persistence was measured from treatment initiation to the end of treatment. Treatment was considered discontinued if the patient died, switched to another anti-fibrotic agent, or failed to receive a prescription of the same anti-fibrotic drug within a predefined permissible gap of 90 days following the end of drug supply.

Medication adherence was estimated based on the Medication Possession Ratio (MPR) metric, which is one of the most widely used metrics for measuring adherence based on administrative data [17,18]. In our case, a fixed duration was assigned to each dispensed package, based on product-specific information: 30 days for nintedanib (packages of 60 capsules of either 150 mg or 300 mg), 28 days for pirfenidone (packages of 252 capsules of 267 mg or 84 capsules of 801 mg), and 14 days for pirfenidone titration packs (63 capsules of 267 mg). The MPR was calculated by dividing the total number of dispensed duration units by the treatment persistence per patient in days. MPR values greater than 1 were truncated to 1, as these reflected early refills.

### 2.6. Statistical Analysis

Continuous variables were summarized with mean along with standard deviation (SD), median along with 1st–3rd quartiles (Q1–Q3) and min–max. Categorical variables were summarized with absolute (n) and relative (%) frequencies. The difference in categorical variables across groups was assessed with Pearson’s Chi-square test or Fisher’s exact test, while a Mann–Whitney statistic test was used for the continuous variables between two groups.

Time on treatment in months was analyzed using Kaplan–Meier methods to estimate the median treatment persistence and corresponding 95% confidence intervals (CIs), along with the rates of patients remaining on treatment at 6, 12, 18, and 24 months, considering deaths and treatment switches as events. To assess whether the observed decline in persistence over time was driven by mortality, persistence rates were additionally estimated with death treated as a censoring event, under both the base-case and gap-based definitions. These results were reported overall and stratified by treatment experience (naive, experienced), sex (male, female), diagnosis (IPF, PPF), and age at treatment initiation (<50, 50–65, 66–75, and ≥76 years). Additional stratified analyses were conducted descriptively within the naive and experienced subgroups. Geographic region was stratified according to the Nomenclature of Territorial Units for Statistics (NUTS) level 1. The log-rank test was used to compare treatment persistence distributions across strata, except where analyses were considered descriptive due to small or selected subgroups.

To explore factors associated with treatment persistence we fitted univariate Cox models and a series of multivariable parametric survival models under both proportional hazards and accelerated failure time (AFT) frameworks. The evaluated parametric distributions included exponential, Weibull, Gompertz, log-normal, log-logistic, and generalized gamma. Model fit across multivariable models was compared using the Akaike Information Criterion and the Bayesian Information Criterion, and the model with the best performance was used for primary inference. Finally, we performed both univariate and multivariable competing risks analyses using the Fine and Gray sub-distribution hazard model, treating death as a competing event. A competing risk is an event whose occurrence precludes the occurrence of another event (treatment switch in base case; switch or temporary interruption in sensitivity analysis). To explore factors associated with MPR, univariate and multivariable fractional logistic regression models were used. Candidate variables with a univariate *p*-value < 0.15 were evaluated for inclusion in the multivariable models, after checking for multicollinearity. All analyses were conducted using STATA version 17.0 (StataCorp, College Station, TX, USA), while Kaplan–Meier survival curves were visualized using the survminer and ggplot2 packages in R version 4.5.1.

## 3. Results

### 3.1. Patient Characteristics

A total of 2112 patients initiated treatment with anti-fibrotic agents during the data-capturing period. Most of them (94%) were treatment-naive, while the rest had prior anti-fibrotic medication experience. The overall cohort was predominantly male (65%), with a median (Q1–Q3) age at treatment initiation of 73 (66–78) years. Most patients were diagnosed with IPF (82%), and nearly all (99%) had at least one documented comorbidity, with gastrointestinal (87%) and cardiovascular (75%) disorders being the most common ones, followed by dyslipidemia (75%). Treatment-experienced patients were more often male (81%) compared with treatment-naive patients (64%) and were older, with 93% aged ≥66 years compared with 76% in the treatment-naive subgroup. In both treatment-naive and treatment-experienced groups, the largest proportion of patients resided in Northern Greece (32% and 39%, respectively). These comparisons are presented for descriptive purposes only (Table 1). Age and sex distribution differed between diagnoses, with 72% vs. 35% being male in IPF and PPF, respectively, while the median age was 74 years (Q1–Q3: 68–78) compared to 66 years (Q1–Q3: 59–73), respectively (Table S3).

### 3.2. Treatment Persistence

**Figure 1:**
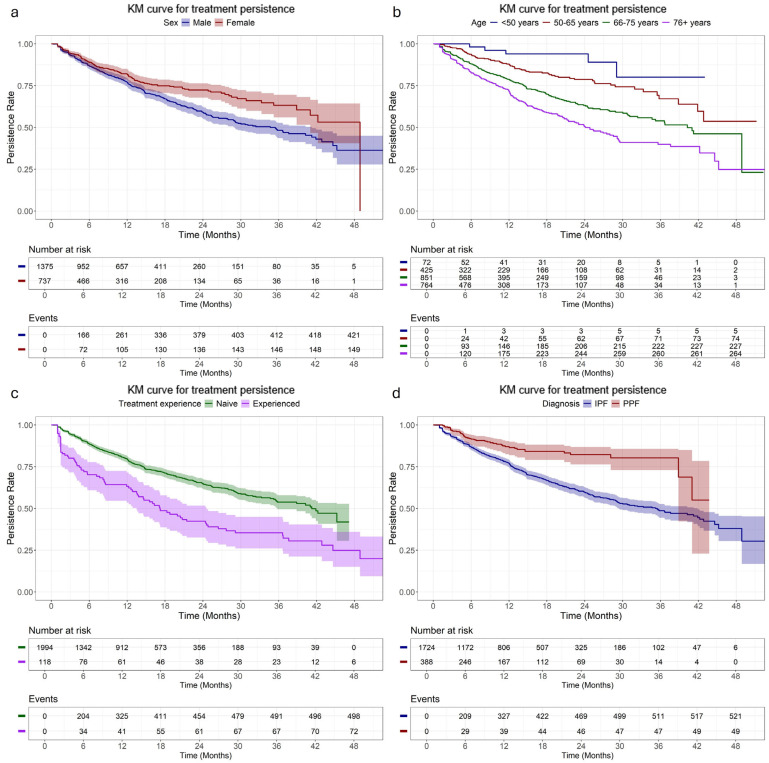
Kaplan-–Meier curve of treatment persistence of patients initiating anti-fibrotic treatment stratified by (**a**) sex, (**b**) age at treatment initiation, (**c**) treatment experience, and (**d**) diagnosis.

**Figure 2:**
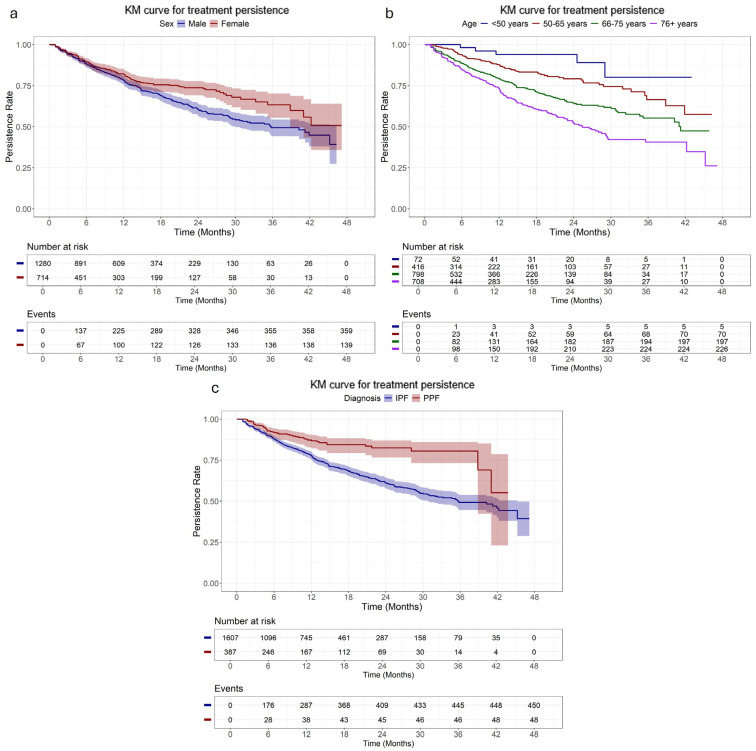
Kaplan-Meier curve of treatment persistence of naive patients initiating anti-fibrotic treatment stratified by (**a**) sex, (**b**) age at treatment initiation, and (**c**) diagnosis.

The overall median treatment persistence was 40.2 months (95% CI: 35.5–44.6), with 79%, and 63% of patients remaining persistent at 12 and 24 months, respectively. Females had a longer median treatment persistence than male patients (48.9 months, 95% CI: 41.0–not reached (NR) vs. 34.7 months, 95% CI: 29.0–41.2; *p* < 0.001), while treatment-naive patients demonstrated a longer median treatment persistence than those with prior treatment experience. Patients aged ≥76 years old had the shortest median persistence (24.1 months, 95% CI: 21.2–29.1) compared to younger groups (*p* < 0.001), with 72% and 50% of them remaining persistent at 12 and 24 months, respectively; the corresponding rates among those aged <50 years old were 94% for both timepoints. The median treatment persistence of patients diagnosed with PPF was not reached and was longer compared to those diagnosed with IPF (median: 35.2 months; 95% CI: 29.3–41.9) (Figure 1, Table 2). Similar findings were observed in the subgroup of treatment-naive patients, with women, younger patients and those with PPF diagnosis experiencing more prolonged treatment persistence compared to their counterparts (Figure 2, Table S4), while among treatment-experienced patients, sex and age did not affect treatment persistence (Figure S1, Table S5).

In sensitivity analyses applying a gap-based definition of treatment persistence (90-day permissible gap), overall treatment duration was shorter, as expected, with a median of 12.6 months (95% CI: 12.0–13.5) (Table S6). Persistence rates declined more rapidly over time, with 53% and 32% of patients remaining persistent at 12 and 24 months, respectively. Despite lower absolute estimates, the overall patterns observed in the base-case analysis were largely preserved, with shorter treatment duration among older patients and among those treated with pirfenidone. Under the gap-based definition, treatment duration was similar between patients with IPF and PPF (median: 12.8 vs. 12.0 months; *p* = 0.948).

When death was treated as a censoring event, persistence rates remained high under the base-case definition but declined substantially under the gap-based definition (Table S7). Counts of observable treatment outcomes, including switching, prolonged treatment interruptions, deaths, and continued treatment at the end of follow-up, are provided in Table S8.

### 3.3. Adherence

The overall mean MPR suggests that patients had maintained medication coverage for approximately 90% of their treatment period. Adherence was the lowest among patients <50 years old and those with prior experience, with mean coverage of 86%. The highest mean MPR was observed among men, patients aged ≥66 years old, treatment-naive patients and those diagnosed with IPF, each with a coverage of 90% (Table 2). Similar patterns were observed in the naive subgroup (Table S4). On the other hand, in the experienced subgroup, adherence was the lowest among patients aged 50 to 65 years, with a median treatment coverage of 73% (Table S5).

### 3.4. Factors Associated with Treatment Persistence

When considering switch and death as a combined event, the results from the multivariable AFT model confirmed KM findings that older age, and IPF diagnosis were associated with a higher risk of treatment switch or death, after adjusting for drug in use and the presence of cardiovascular and gastrointestinal disorders. Particularly, patients aged ≥76 years old had an 82% reduction in time-to-switch or death compared to those <50 years old [adjusted time ratio (TR) = 0.18, 95% CI: 0.09–0.39, *p* < 0.001)] (Table 3).

When considering switch as the primary event and death as a competing risk, in the multivariable competing risk model, IPF diagnosis and the presence of gastrointestinal disorders were the only groups associated with higher risk of discontinuation. In addition, regional differences were observed, with patients residing in Aegean and Crete having a higher hazard of treatment switching compared with those residing in Attica. The associations for age observed in the AFT model were diminished and did not reach statistical significance, indicating that the increased risk of discontinuation in older patients was primarily driven by higher mortality rather than treatment switching alone. Sex and drug variables were not associated with treatment discontinuation in the univariate models, resulting in their exclusion from the multivariable analysis (Table 3).

In sensitivity analyses applying a gap-based definition of treatment persistence, the main patterns observed in the primary analyses were largely confirmed. In the accelerated failure time models, treatment persistence remained shorter with increasing age and among patients treated with pirfenidone, although associations for diagnosis were attenuated. In competing risk models focusing on treatment switching with death as a competing event, age was no longer associated with switching, further supporting that the age-related effects observed in the primary analysis were largely driven by mortality. Regional associations observed in the primary competing risk analysis were not confirmed in sensitivity analyses (Table S10).

### 3.5. Factors Associated with Adherence

Results from the multivariable fractional logistic regression model suggest that female sex, PPF diagnosis, use of pirfenidone, and residence in Northern Greece and Central Greece (compared with Attica) are associated with reduced treatment adherence, while older age is linked to improved adherence. The presence of neurological disorders and infections was linked with reduced adherence in the univariate models, but these associations did not reach statistical significance in the multivariable model (Table 4).

## 4. Discussion

This study presents unique data concerning the treatment persistence rates of anti-fibrotics for the Greek territory. Having the opportunity to exploit data derived from the national prescription database, we were able to construct a cohort which to our knowledge constitutes the largest cohort applied in similar studies so far. Moreover, this is the first study to provide persistence rates for anti-fibrotic regimens for ILDs other than IPF.

We observed an overall treatment persistence of 78.7% for the first year. This finding is within the margins recorded by previous reports employing patient-based registries [9,10]. Although we are not aware of the individual reasons for discontinuation, an approximate discontinuation rate of 20% during the first year closely resembles the rates observed in the key clinical trials of both anti-fibrotic drugs [3,4] and in real-world evidence studies [10,11,12].

The persistence rates declined gradually across subgroups. Particularly for the second year we observed an overall persistence rate of 63.2%. The median age of patients included in our analysis is among the highest reported in similar retrospective studies [8,10,11,12,16] suggesting a potential effect of age. The lowest persistence rates observed for the elderly patients across all subgroups longitudinally compared to the younger counterparts and the higher risk of treatment switch or death corroborate this notion [19,20,21]. Alternatively, increased dropping rates of anti-fibrotics could be due to the combinatorial effect of mortality. Of note, a 3-year mortality rate ranging from 27% to 37% has been reported in retrospective real-world studies assessing the efficacy of anti-fibrotic drugs in the Greek population [14,22]. Our analysis indicates that mortality is significantly associated with the decline of persistence rates observed during the second year in the base-case scenario, but sensitivity analysis demonstrated that reduced persistence over time was not solely attributable to mortality but also to clinical reasons.

Individual clinical data concerning the lung function [21], performance status [8] and the burden of adverse events [23] among others may be associated with reduced treatment persistence. Unfortunately, the database that we had access did not contain this information. Therefore, the impact of these factors could be further assessed in future studies to elaborate further on our findings.

Higher persistence rates in female patients compared to their male counterparts were observed, although sex was not detected as a significant factor for increased persistence. Although women represent a smaller fraction in similar retrospective studies, possibly due to underdiagnosis [24], their quality of life seems to be impaired in a lesser extent [25] while they demonstrate slower progression of the disease [26] compared to men. Additionally, women have been shown to have better survival and tolerability following dose adjustments compared to men [27]. This gender-specific difference could be based upon the less advanced disease burden at diagnosis and better lung function that women patients may tend to present [28]. This could be attributed to their tendency to seek primary healthcare more often compared to men, possibly due to a lower treatment-seeking threshold [29]. These could be some factors justifying observed increased persistence rates in women, although female sex was associated with reduced treatment adherence. Therefore, further studies are warranted to unravel a possible interaction between adherence and persistence and whether anatomical or physiological differences confer to the sex-related differences.

Shorter treatment persistence was observed among patients with prior anti-fibrotic treatment experience. This finding is contradictory to studies stating that switching to a second anti-fibrotic agent improves overall survival and prognosis of IPF patients [7,30]. However, this finding warrants further confirmation and should be interpreted with caution given the small and heterogeneous nature of this subgroup.

We show that IPF diagnosis is associated with a higher risk of discontinuation compared to the PPF subgroup. Patients with IPF diagnosis dominated our cohort, and therefore this result should be interpreted with caution. It is interesting that SSc-ILD diagnosis, which falls in the PPF subgroup, more often occurs in young female patients [31]. This is supported by our cohort, whereas the ratio of men to women was reversed in the subgroup of PPF diagnosis, while the median age at treatment initiation was also significantly lower. Given our previous findings, the existence of younger patients and more women would contribute to an increase in the observed persistence rates of nintedanib therapy.

We further observed that patients with gastrointestinal disorders have a higher risk of discontinuation when death was considered as a competing risk. Gastro-oesophageal reflux disease is a very common comorbidity of IPF patients, with a reported prevalence of up to 60%, while a substantial fraction of patients were expected to remain asymptomatic [32]. Additionally, gastrointestinal events have been reported to be the most common adverse reactions observed in the Greek population following anti-fibrotic medication administration [14,16]. This finding should raise caution towards the improvement of adverse reaction management in order for the discontinuation rates to remain in low levels.

Although it could be speculated that older patients would have reduced medication adherence, we observed higher MPR indices for aged patients compared to their younger counterparts. This could be attributed to improved therapeutic protocols that entail constant therapeutic monitoring [33] or to family and social support that have been shown to be highly effective in ensuring that elder patients consume their medications [34]. Social support tends to be provided in a more organized way in rural areas compared to urban areas [35]. However, rural areas in Greece are underserved in terms of healthcare infrastructure and healthcare human resources that are mainly concentrated in widened metropolitan areas of big cities such as Athens [36]. This uneven distribution, on the contrary, may lead to reduced medication adherence [37]. Indeed, using the residence of the prescribing physician as a proxy in our analysis, we observed a reduced adherence rate for patients residing in rural areas. This controversy warrants further research and is essential for the efforts of the local authorities to improve strategies aiming to improve medication adherence. Such efforts should also incorporate the unique characteristics of each geographic area, such as ethnic and racial disparities [38,39] as well as the prevalence of occupational and professional hazards [40] that have an impact on medication adherence and prevalence of fibrotic diseases. Unfortunately, we did not have access to individual patient data concerning their residence, income classification and ethnic group, thus limiting our ability to fully delineate the impact of socio-economic status in the adherence ratio.

As a retrospective observational analysis, several limitations apply. Our results are subject to residual confounding and unmeasured biases, despite adjustment for multiple patient characteristics. Because the database does not record clinical reasons for treatment discontinuation, switching and prolonged interruptions should be interpreted as observable treatment patterns rather than direct indicators of adverse events. Treatment assignment was not randomized, and clinical factors such as disease severity or physician preference may have influenced both drug choice and treatment persistence. Adherence was assessed using MPR, which, although widely used in pharmacoepidemiologic studies, may overestimate true adherence as it does not capture actual medication uptake or dose skipping, which is a limitation that is particularly relevant for anti-fibrotic therapies frequently associated with gastrointestinal adverse effects [3,4]. Furthermore, the grouping of diverse ILD entities under the PPF category represents an additional limitation, as these conditions differ in terms of underlying pathophysiology, clinical course, and treatment response [41]. However, subgroup-specific analyses (e.g., RA-ILD, SSc-ILD, unclassifiable PF) were not feasible due to limited sample sizes, and this heterogeneity would have influenced the observed persistence and adherence estimates. Comorbidities were captured as binary indicators by category, as the data source did not allow construction of validated comorbidity indices (e.g., Charlson Comorbidity Index) or assessment of disease severity; therefore, residual confounding related to overall health status cannot be excluded. Competing risks were handled using the Fine and Gray method, which assumes correct classification of competing events (e.g., switch vs. death). Misclassification or non-detected reasons for treatment discontinuation could bias the estimated sub-distribution hazard ratios.

## 5. Conclusions

Collectively, we have generated real-world evidence concerning the utilization of anti-fibrotic drugs in Greece. The persistence rates for the first year were generally consistent with what has been reported elsewhere. Elderly patients with an IPF diagnosis as well as treatment-experienced patients and those with an increased burden of comorbidities seem to have increased odds to discontinue anti-fibrotic therapy. Our findings highlight the importance of adverse reactions management. This way, the persistence of patients in a therapeutic scheme based on anti-fibrotic drugs might be enabled, thus leading to the optimization of therapeutic management of ILDs. This is very important, particularly for elderly patients and those that need to switch to a different anti-fibrotic regimen.

## Figures and Tables

**Table 1:** Patient characteristics of patients initiating anti-fibrotic treatment overall and stratified by treatment experience.

		Naive	Experienced
Patient Characteristics	N = 2112	N = 1994	N = 118
*Sex, n (%)*			
Male	1375 (65.1)	1280 (64.2)	95 (80.5)
Female	737 (34.9)	714 (35.8)	23 (19.5)
*Age at treatment initiation, n (%)*			
<50 years	72 (3.4)	72 (3.6)	0 (0)
50–65 years	425 (20.1)	416 (20.9)	9 (7.6)
66–75 years	851 (40.3)	798 (40.0)	53 (44.9)
76+ years	764 (36.2)	708 (35.5)	56 (47.5)
*Age at treatment initiation, in years*			
Mean (SD)	71.0 (9.7)	70.8 (9.8)	75.0 (6.5)
Median (Q1–Q3)	73 (66–78)	72 (66–77)	75 (70–80)
Min–max	21–91	21–91	56–89
*Diagnosis, n (%)*			
IPF	1724 (81.6)	1607 (80.6)	117 (99.2)
PPF	388 (18.4)	387 (19.4)	1 (0.9)
*Region (NUTS1), n (%)*			
Attica	540 (25.8)	529 (26.7)	11 (9.3)
Aegean and Crete	279 (13.3)	234 (11.8)	45 (38.1)
North Greece	680 (32.4)	634 (32.0)	46 (39.0)
Central Greece	598 (28.5)	582 (29.4)	16 (13.6)
*Comorbidities, n (%)*	**N = 2088**	**N = 1971**	**N = 117**
At least one	2063 (98.8)	1947 (98.8)	116 (99.2)
Gastrointestinal disorders	1824 (87.4)	1723 (87.4)	101 (86.3)
Cardiovascular disorders	1569 (75.1)	1474 (74.8)	95 (81.2)
Dyslipidemia	1564 (74.9)	1478 (75.0)	86 (73.5)
Diabetes mellitus	696 (33.3)	668 (33.9)	28 (23.9)
Osteoporosis	505 (24.2)	483 (24.5)	22 (18.8)
Hyperuricemia	461 (22.1)	432 (21.9)	29 (24.8)
Infections	233 (11.2)	219 (11.1)	14 (12.0)
Rheumatological disorders	232 (11.1)	226 (11.5)	6 (5.1)
Neurological disorders	45 (2.2)	40 (2.0)	5 (4.3)
Psoriasis	33 (1.6)	30 (1.5)	3 (2.6)
Psychiatric disorders	13 (0.6)	13 (0.7)	0 (0.0)
Thromboembolic diseases	11 (0.5)	11 (0.6)	0 (0.0)

Abbreviations: IPF: idiopathic pulmonary fibrosis; NUTS1: Nomenclature of Territorial Units for Statistics level 1; PPF: progressive pulmonary fibrosis; Q1–Q3: 1st–3rd quartile; SD: standard deviation.

**Table 2:** Treatment persistence (in months) of anti-fibrotic treatment, persistence rates, and medication possession ratio overall and stratified by sex, age at treatment initiation, treatment experience, and diagnosis.

Treatment Persistence in Months	Median (95% CI)	*p*-Value ^1^	% Persistent at				MPR		
			6 Months	12 Months	18 Months	24 Months	Mean (SD)	Median (Q1–Q3)	Min–Max
**Overall**	40.2 (35.5, 44.6)		87.7%	78.7%	69.6%	63.2%	0.90 (0.14)	0.95 (0.85–1.00)	0.18–1.00
*Sex*									
Male	34.7 (29.0, 41.2)	**<0.001**	87.0%	77.1%	67.0%	59.0%	0.90 (0.13)	0.95 (0.87–1.00)	0.22–1.00
Female	48.9 (41.0, NR)		89.0%	81.9%	74.9%	72.4%	0.89 (0.15)	0.96 (0.83–1.00)	0.18–1.00
*Age at treatment initiation*									
<50	NR (NR, NR)	**<0.001**	98.2%	94.0%	94.0%	94.0%	0.86 (0.16)	0.92 (0.77–1.00)	0.48–1.00
50–65	NR (41.9, NR)		93.8%	88.0%	82.7%	78.7%	0.89 (0.15)	0.95 (0.84–1.00)	0.18–1.00
66–75	40.2 (34.7, NR)		88.1%	78.8%	69.9%	63.2%	0.90 (0.14)	0.96 (0.86–1.00)	0.22–1.00
76+	24.1 (21.2, 29.1)		82.8%	71.7%	58.8%	50.4%	0.90 (0.13)	0.95 (0.86–1.00)	0.32–1.00
*Treatment experience*									
Naive	41.2 (35.8, NR)		88.7%	79.6%	71.1%	64.8%	0.90 (0.14)	0.95 (0.85–1.00)	0.18–1.00
Experienced	17.2 (13.5, 24.6)		70.2%	63.3%	48.6%	42.3%	0.86 (0.16)	0.93 (0.79–0.98)	0.32–1.00
*Diagnosis*									
IPF	35.2 (29.3, 41.9)	**<0.001**	86.8%	77.0%	66.7%	59.7%	0.90 (0.13)	0.95 (0.86–1.00)	0.21–1.00
PPF	NR (38.8, NR)		91.5%	87.0%	84.2%	82.2%	0.89 (0.15)	0.96 (0.81–1.00)	0.18–1.00

Note: ^1^ Log-rank test. Abbreviations: CI: confidence interval; IPF: idiopathic pulmonary fibrosis; MPR: medication possession ratio; NR: not reached; PPF: progressive pulmonary fibrosis; Q1–Q3: 1st–3rd quartile; SD: standard deviation.

**Table 3:** Associations between patient characteristics and treatment persistence: results from accelerated failure time and competing risks regression models.

	Generalized Gamma AFT Regression				Fine and Gray Competing Risk Regression			
	Univariate Model		Multivariable Model		Univariate Model		Multivariable Model	
	N = 2112		N = 2088		N = 2112		N = 2088	
	TR (95% CI)	*p*-Value	TR (95% CI)	*p*-Value	SHR (95% CI)	*p*-Value	SHR (95% CI)	*p*-Value
*Sex*								
Female vs. Male	1.37 (1.12, 1.67)	**0.002**	1.12 (0.91, 1.37)	0.274	0.97 (0.71, 1.32)	0.828		
*Age at treatment initiation*								
50–65 years vs. <50 years	0.41 (0.20, 0.86)	**<0.001**	0.47 (0.22, 1.00)	**<0.001**	2.20 (0.53, 9.19)	**0.043**	1.64 (0.39, 6.86)	0.305
66–75 years vs. <50 years	0.22 (0.11, 0.45)		0.26 (0.12, 0.55)		3.57 (0.88, 14.4)		2.35 (0.57, 9.65)	
76+ years vs. <50 years	0.15 (0.07, 0.31)		0.18 (0.09, 0.39)		3.52 (0.87, 14.3)		2.19 (0.53, 9.03)	
*Diagnosis*								
PPF vs. IPF	2.19 (1.66, 2.88)	**<0.001**	1.44 (1.07, 1.94)	**0.015**	0.24 (0.12, 0.48)	**<0.001**	0.29 (0.14, 0.59)	**0.001**
*Drug*								
Pirfenidone vs. Nintedanib	0.60 (0.48, 0.73)	**<0.001**	0.73 (0.59, 0.90)	**0.003**	0.83 (0.58, 1.19)	0.306		
*Comorbidities, yes vs. no*					N = 2088			
Cardiovascular disorders	0.74 (0.60, 0.92)	**0.007**	1.04 (0.83, 1.31)	0.709				
Gastrointestinal disorders					1.94 (1.11, 3.40)	**0.021**	2.08 (1.19, 3.66)	**0.010**
*Region (NUTS1)*	N = 2097				N = 2097			
Aegean and Crete vs. Attica	0.68 (0.50, 0.92)	0.088	0.80 (0.59, 1.08)	0.463	2.21 (1.39, 3.51)	**0.003**	1.83 (1.13, 2.94)	**0.018**
Northern Greece vs. Attica	0.85 (0.66, 1.09)		0.95 (0.74, 1.22)		1.78 (0.76, 1.82)		0.99 (0.63, 1.55)	
Central Greece vs. Attica	0.80 (0.61, 1.03)		0.88 (0.67, 1.14)		1.49 (0.96, 2.30)		1.23 (0.79, 1.92)	

Notes: *p*-value for “age at treatment initiation” was derived from an overall Wald test. Selection of comorbidities was based on the univariate models presented in Table S9. The event for AFT models was switch or death as combined event. The primary event for competing risk analysis was switch with death as competing risk. Variables with *p* < 0.15 in the univariate level were entered in the multivariable models. Proportionality of hazards was violated for treatment experience and drug. The generalized gamma AFT was selected, among other parametric survival models, based on AIC and BIC. In the generalized gamma acceleration AFT model, the coefficients are interpreted in terms of TRs rather than hazard ratios. The TR is derived as TR = eCoefficient, which represents the factor by which the time to the event changes per unit increase in the covariate. A TR < 1 indicates shorter time to the event (higher risk), and a TR > 1 indicates longer time to the event (lower risk). SHR focus on how covariates influence the probability of a specific event (e.g., switch) while accounting for the competing event (e.g., death). The SHR reflects the risk of the primary event in the presence of competing risks, not a combined outcome. An SHR > 1 indicates an increased hazard (higher risk of the event), while an SHR < 1 indicates a decreased hazard (lower risk of the event). Abbreviations: AFT: acceleration failure time; CI: confidence interval; IPF: idiopathic pulmonary fibrosis; NUTS1: Nomenclature of Territorial Units for Statistics level 1; PPF: progressive pulmonary fibrosis; SHR: Sub-distribution Hazard Ratios; TR: time ratio.

**Table 4:** Associations between patient characteristics and treatment adherence: results from univariate and multivariable fractional logistic regressions.

	Univariate Fractional Logistic Regression		Multivariable Fractional Logistic Regression	
	N = 2112		N = 2088	
Treatment Adherence	OR (95% CI)	*p*-Value	OR (95% CI)	*p*-Value
*Sex*				
Female vs. Male	0.84 (0.73, 0.96)	**0.013**	0.83 (0.72, 0.96)	**0.013**
*Age at treatment initiation*				
50–65 years vs. <50 years	1.31 (0.94, 1.83)	**0.049**	1.31 (0.93, 1.83)	**0.041**
66–75 years vs. <50 years	1.48 (1.08, 2.04)		1.49 (1.08, 2.07)	
76+ years vs. <50 years	1.49 (1.08, 2.05)		1.53 (1.10, 2.12)	
*Diagnosis*				
PPF vs. IPF	0.84 (0.71, 0.99)	**0.038**	0.78 (0.64, 0.94)	**0.011**
*Drug*				
Pirfenidone vs. Nintedanib	0.64 (0.56, 0.73)	**<0.001**	0.58 (0.50, 0.67)	**<0.001**
*Comorbidities, yes vs. no*	N = 2088			
Neurological disorders	0.69 (0.48, 0.99)	**0.043**	0.70 (0.49, 1.01)	0.057
Infections	0.79 (0.65, 0.97)	**0.023**	0.83 (0.68, 1.02)	0.084
*Region (NUTS1)*	N = 2097			
Aegean and Crete vs. Attica	0.996 (0.78, 1.24)	**0.009**	0.87 (0.69, 1.09)	**0.022**
Northern Greece vs. Attica	0.79 (0.67, 0.94)		0.76 (0.64, 0.91)	
Central Greece vs. Attica	0.79 (0.67, 0.95)		0.81 (0.67, 0.96)	

Notes: *p*-value for “age at treatment initiation” was derived from an overall Wald test. Selection of comorbidities was based on the univariate models presented in Table S6. An OR < 1 indicates that the given category is associated with lower odds of greater adherence compared to the reference group, while an OR > 1 indicates that the given category is associated with higher odds of greater adherence compared to the reference group. Abbreviations: CI: confidence interval; IPF: idiopathic pulmonary fibrosis; NUTS1: Nomenclature of Territorial Units for Statistics level 1; OR: odds ratio; PPF: progressive pulmonary fibrosis.

## Data Availability

Data concerning this manuscript is available from the corresponding author upon reasonable request.

## Supplementary Materials

The following supporting information can be downloaded at https://www.mdpi.com/article/10.3390/arm94010006/s1, Figure S1: Kaplan–Meier curve of treatment persistence of experienced patients initiating anti-fibrotic treatment stratified by (a) sex, (b) age at treatment initiation, and (c) diagnosis; Table S1: Diagnostic Classification Based on ICD-10 Codes; Table S2: Therapeutic health protocols classification; Table S3: Patient characteristics of patients initiating anti-fibrotic treatment stratified by diagnosis; Table S4: Treatment persistence (in months) of anti-fibrotic treatment, persistence rates and medication possession ratio overall and stratified by sex, age at treatment initiation and diagnosis in naive patients initiating anti-fibrotic treatment; Table S5: Treatment persistence (in months) of anti-fibrotic treatment, persistence rates and medication possession ratio overall and stratified by sex, age at treatment initiation and diagnosis in experienced patients initiating anti-fibrotic treatment; Table S6: Treatment duration of the first observed treatment overall and stratified by drug, sex and sub-population in patients initiating anti-fibrotic treatment (Sensitivity analysis); Table S7: Treatment persistence rates with death treated as a censoring event, under the base-case and gap-based (90-day) definitions, overall and stratified by sex, age at treatment initiation, and diagnosis; Table S8: Observable reasons for treatment cessation based on prescription patterns; Table S9: Supporting information in variables selection for multivariable survival models and multivariable fractional regression models (base-case analysis). Results from univariate models regarding comorbidities; Table S10: Associations between patient characteristics and treatment persistence: results from accelerated failure time, and competing risks regression models (Sensitivity analysis).

## Abbreviations

The following abbreviations are used in this manuscript:

AFT	Accelerated Failure Time
CIs	Confidence Intervals
CTD-ILD	Connective Tissue Disease—ILD
FVC	Forced Vital Capacity
ICD-10	International Statistical Classification of Diseases and Related Health Problems 10th Revision
IDIKA	National Electronic Prescription Database
ILDs	Interstitial Lung Diseases
IPF	Idiopathic Pulmonary Fibrosis
KM	Kaplan–Meier
MPR	Medication Possession Ratio
NR	Not Reached
NUTS-1	Nomenclature of Territorial Units for Statistics-1
PF	Pulmonary Fibrosis
PPF	Progressive Pulmonary Fibrosis
Q1-Q3	1st–3rd quartile
RA-ILD	Rheumatoid Arthritis-associated ILD
SD	Standard Deviation
SLE-ILD	Systemic Lupus Erythematosus associated ILD
SSc-ILD	Systemic Sclerosis-associated ILD

## Appendix A. Diagnosis Definition

The diagnosis classification was based on the ICD-10 codes of the prescriptions received during the data capturing period. Τhe ICD-10 codes extracted from each prescription were grouped into ten diagnoses based on the classification outlined in Table S1. Diagnosis of IPF was defined using the ICD-10 code J84. Each prescription could contain more than one ICD-10 code and as such, each prescription could be linked with one or more diagnoses.

Patients were assigned to an IPF diagnosis if (a) all prescriptions executed during the follow-up period were linked only with the IPF diagnosis, (b) IPF was the most frequently recorded diagnosis among all executed prescriptions, either as the sole diagnosis or in combination with other diagnoses, and if (c) IPF was tied with other diagnoses, but was prioritized as the most severe condition according to a predefined hierarchical classification system: IPF > systemic sclerosis-associated (SSc)-ILD > rheumatoid arthritis-associated (RA)-ILD > systemic lupus erythematosus > dermatomyositis–polymyositis > Sjogren’s syndrome > unclassifiable PF > myositis > other PF-ILDs.
