# Supplementary material for: Treatment Adherence and Persistence of Anti-Fibrotic Drugs in Real Life in Greece
Source: Adv Respir Med. 2026 Jan 8;94(1):6. doi: 10.3390/arm94010006 (PMC12821729; doi:10.3390/arm94010006)
Supplement: Supplementary file 1 [file arm-94-00006-s001.zip › arm-3956323-supplementary.pdf]

Supplementary material

Supplementary Tables

Table S1. Diagnostic Classification Based on ICD-10 Codes.

**Diagnosis**	**ICD-10 code**
Idiopathic Pulmonary Fibrosis (IPF)	J84.1
Unclassifiable Pulmonary Fibrosis (PF)	J84.9
Rheumatoid Arthritis (RA)-Associated Interstitial Lung Disease (ILD)	J99, J99.0, M05, M05.1, M05.3, M06.0, M06.9, M08.0, M13, M13.9
Systemic Sclerosis (SSc)-Associated ILD	J99.1, M34, M34.0, M34.8, M34.9, M34.1, M35.2, M35.8, M35.9
Systemic Lupus Erythematosus (SLE)-Associated ILD	M32, M32.1, M32.9
Sjogren Syndrome	M35.0
Dermatomyositis	M33.1
Polymyositis	M33, M33.2, M33.9
Myositis	M60, M60.8, M60.9
Other PF-ILDs	J84, I27.0, J68.4, J84.8, M35.1, D86, J67.9, J45.0, M06, M31.7, U07.1, T88.7, I27.2, C34, J44, M81.5, J45.9, P83.8, J45, M35, M80.0, M81.4, E84, G73.7, I26, J68, M07, M31.3, Z25.1, D86.0, L94.0, J17.0, J67.8
Tie-Breaking Hierarchy: If multiple diagnoses had equal proportions, classification followed a predefined hierarchy, prioritizing more severe diseases: IPF > SSc-ILD > RA-ILD > SLE > Dermatomyositis-Polymyositis > Sjogren > Unclassifiable PF > Myositis > Other PF-ILDs.	

Table S2. Therapeutic health protocols classification.

**Classification**	**Therapeutic Health Protocols**[a]
Respiratory disorders	Bronchitis
	Acute exacerbation of Chronic Obstructive Pulmonary Disease (COPD)
	Chronic Obstructive Pulmonary Disease (COPD)
Gastrointestinal disorders	Gastroesophageal reflux disease (GERD)
	Gastroprotection
	Dyspepsia
	Eradication of Helicobacter pylori
	Ulcerative colitis
	Crohn's disease
	Peptic ulcer
Dyslipidemia	Dyslipidemia - Administration of PCSK-9 inhibitors
	Dyslipidemia
Cardiovascular disorders	Arterial hypertension
	Heart failure
	Atrial fibrillation
	Coronary artery disease
Infections	Infectious gastroenteritis
	Upper urinary tract infections
	Lower urinary tract infections (uncomplicated/complicated)
	Acute rhinosinusitis
	Acute pharyngotonsillitis

Classification	Therapeutic Health Protocols[a]
	Community-acquired pneumonia
Neurological disorders	Multiple sclerosis with predominantly relapsing/remitting course
	Advanced Parkinson's disease
	Early-onset Parkinson's disease
	Other generalized epilepsy and syndromes
	Generalized epilepsy
	Focal epilepsy
	Alzheimer's disease
Paget's disease of bones	Paget's disease of bones
Osteoporosis	Men - With or without fracture
	Women - With hip fracture
	Women - With non-spinal fracture
	Women - With spinal fracture
	Women - Without fracture
	High risk of low-trauma fractures
Rheumatological disorders	Axial spondyloarthritis (radiographically confirmed or not)
	Gout - Treatment and prevention of attacks
	Established or early rheumatoid arthritis
	Psoriatic arthritis (Peripheral involvement)
Diabetes mellitus	Neonatal diabetes mellitus
	Gestational diabetes mellitus
	Type 1 diabetes mellitus (insulin-dependent diabetes mellitus)
	Type 2 diabetes mellitus
	Diabetes mellitus - Insulin deficiency following medical procedures
	Other types of diabetes mellitus
Hyperuricemia	Hyperuricemia
Thromboembolic diseases	Cancer treatment
	Venous thromboembolic disease treatment
	Prevention in cancer patients
	Prevention in orthopedic trauma and burn patients
	Prevention and treatment in pregnancy and puerperium - vascular disorders of the placenta - by six weeks post-abortion
	Prevention in hospitalized patients with acute non-surgical illness
	Prevention in surgical patients without malignancy
Psychiatric disorders	Generalized anxiety disorder
	Social anxiety disorder
	Panic disorder
	Obsessive-compulsive disorder
	Mild depression
	Recurrent and chronic depression

Notes: [a]The table is taken from Greek Ministry of Health (https://www.moh.gov.gr, accessed 31st of June 2026). Abbreviations: PCSK: proprotein convertase subtilisin/kexin type 9.

Table S3. Patient characteristics of patients initiating anti-fibrotic treatment stratified by diagnosis.

Patient characteristics	IPF N=1,724	PPF N=388	P
Sex, n (%)			
Male	1,238 (71.8)	137 (35.3)	**<0.001**
Female	486 (28.2)	251 (64.7)	
Age at treatment initiation, n (%)			
< 50 years	30 (1.7)	42 (10.8)	**<0.001**
50 – 65 years	280 (16.2)	145 (37.4)	
66 – 75 years	728 (42.2)	123 (31.7)	
76+ years	686 (39.8)	78 (20.1)	
Age at treatment initiation, in years			
Mean (SD)	72.4 (8.5)	65.0 (11.8)	
Median (Q1 – Q3)	74 (68 – 78)	66 (59 – 73)	**<0.001**[a]
Min – max	21 – 91	23 – 89	
Region (NUTS1), n (%)	**N=1,710**	**N=387**	
Attica	346 (20.2)	194 (50.1)	**<0.001**
Aegean & Crete	252 (14.7)	27 (7.0)	
North Greece	583 (34.1)	97 (25.1)	
Central Greece	529 (30.9)	69 (17.8)	
Comorbidities, n (%)	**N=1,704**	**N=384**	
At least one	1,682 (98.7)	381 (99.2)	
Gastrointestinal disorders	1465 (86.0%)	359 (93.5%)	**<0.001**
Cardiovascular disorders	1319 (77.4%)	250 (65.1%)	**<0.001**
Dyslipidemia	1319 (77.4%)	245 (63.8%)	**<0.001**
Diabetes mellitus	597 (35.0%)	99 (25.8%)	**0.001**
Osteoporosis	347 (20.4%)	158 (41.1%)	**<0.001**
Hyperuricemia	417 (24.5%)	44 (11.5%)	**<0.001**
Infections	182 (10.7%)	51 (13.3%)	0.144
Rheumatological disorders	137 (8.0%)	95 (24.7%)	**<0.001**
Neurological disorders	41 (2.4%)	4 (1.0%)	0.096
Psoriasis	32 (1.9%)	1 (0.3%)	0.022
Psychiatric disorders	11 (0.6%)	2 (0.5%)	0.779
Thromboembolic diseases	9 (0.5%)	2 (0.5%)	0.986

Note: P-values were derived from Pearson's chi squared tests, except otherwise specified. a: Mann-Whitney test. Abbreviations: IPF: idiopathic pulmonary fibrosis; PPF: progressive pulmonary fibrosis; Q1-Q3: 1st-3rd quartile; SD: standard deviation.

Table S4. Treatment persistence (in months) of anti-fibrotic treatment, persistence rates and medication possession ratio overall and stratified by sex, age at treatment initiation and diagnosis in naïve patients initiating anti-fibrotic treatment.

Treatment persistence in months	Median (95% CI)	p-value	% persistent at 6 months	12 months	18 months	24 months	MPR Mean (SD)	Median (Q1 – Q3)	Min – Max
**Overall**	41.2 (35.8, NR)		88.7%	79.6%	71.1%	64.8%	0.90 (0.14)	0.95 (0.85 – 1.00)	0.18 – 1.00
Sex									
Male	35.8 (30.0, NR)	**0.002**	88.4%	78.4%	68.9%	60.5%	0.91 (0.13)	0.95 (0.88 – 1.00)	0.22 – 1.00
Female	NR (38.8, NR)		89.4%	82.0%	75.6%	73.7%	0.89 (0.15)	0.96 (0.83 – 1.00)	0.18 – 1.00
Age at treatment initiation									
< 50	NR (NR, NR)	**< 0.001**	98.2%	94.0%	94.0%	94.0%	0.86 (0.16)	0.92 (0.77 – 1.00)	0.48 – 1.00
50 – 65	NR (41.9, NR)		93.9%	87.9%	83.3%	79.2%	0.89 (0.14)	0.95 (0.84 – 1.00)	0.18 – 1.00
66 – 75	41.2 (35.2, NR)		88.7%	79.4%	71.1%	64.6%	0.91 (0.14)	0.96 (0.86 – 1.00)	0.22 – 1.00
76+	25.4 (22.4, 29.4)		84.7%	73.2%	60.5%	52.1%	0.90 (0.13)	0.95 (0.86 – 1.00)	0.35 – 1.00
Diagnosis									
IPF	35.6 (30.8, 45.2)	**< 0.001**	88.0%	78.0%	68.3%	61.2%	0.90 (0.13)	0.95 (0.87 – 1.00)	0.21 – 1.00
PPF	NR (38.8, NR)		91.8%	87.3%	84.5%	82.5%	0.89 (0.15)	0.96 (0.81 – 1.00)	0.18 – 1.00

Note: P-values were derived from log-rank tests. Abbreviations: CI: confidence interval; IPF: idiopathic pulmonary fibrosis; MPR: Medication Possession Ratio; NR: not reached; PPF: progressive pulmonary fibrosis; Q1-Q3: 1st-3rd quartile; SD: standard deviation.

Table S5. Treatment persistence (in months) of anti-fibrotic treatment, persistence rates and medication possession ratio overall and stratified by sex, age at treatment initiation and diagnosis in experienced patients initiating anti-fibrotic treatment.

Treatment persistence in months	Median (95% CI)	p-value	% persistent at 6 months	12 months	18 months	24 months	MPR Mean (SD)	Median (Q1 – Q3)	Min – Max
**Overall**	17.2 (13.5, 24.6)		70.2%	63.3%	48.6%	42.3%	0.86 (0.16)	0.93 (0.79 – 0.98)	0.32 – 1.00
Sex									
Male	17.1 (11.9, 24.6)	0.363	69.0%	60.6%	46.7%	41.7%	0.87 (0.16)	0.93 (0.80 – 0.97)	0.32 – 1.00
Female	20.7 (12.3, NR)		76.3%	76.3%	58.7%	45.7%	0.86 (0.18)	0.95 (0.77 – 1.00)	0.35 – 1.00
Age at treatment initiation									
< 50									
50 – 65	42.8 (1.0, NR)	0.101	88.9%	88.9%	63.5%	63.5%	0.73 (0.20)	0.75 (0.53 – 0.92)	0.46 – 0.95
66 – 75	19.9 (13.8, 36.6)		78.5%	69.5%	55.1%	47.9%	0.86 (0.17)	0.93 (0.77 – 0.97)	0.35 – 1.00
76+	13.5 (4.7, 20.7)		59.4%	53.2%	40.2%	33.5%	0.90 (0.13)	0.93 (0.85 – 1.00)	0.32 – 1.00
Diagnosis									
IPF	17.2 (13.5, 24.6)		70.9%	63.8%	49.1%	42.7%	0.87 (0.16)	0.93 (0.80 – 0.98)	0.32 – 1.00
PPF	-		-	-	-	-	0.77 (.)		

Note: P-values were derived from log-rank tests. Abbreviations: CI: confidence interval; IPF: idiopathic pulmonary fibrosis; MPR: Medication Possession Ratio; NR: not reached; PPF: progressive pulmonary fibrosis; Q1-Q3: 1st-3rd quartile; SD: standard deviation.

Table S6. Treatment duration of the first observed treatment overall and stratified by drug, sex and sub-population in patients initiating anti-fibrotic treatment (Sensitivity analysis).

Treatment duration	No of patients	Median (95% CI)	p-value	% persistent at 6 months	% persistent at 12 months	% persistent at 18 months	% persistent at 24 months
**Overall**	2,112[a]	12.6 (12.0, 13.5)		70.8%	52.5%	40.4%	32.2%
Sex							
Male	1,375	13.4 (12.5, 14.6)	**0.020**	72.2%	54.8%	42.1%	33.7%
Female	737	11.6 (10.0, 12.5)		68.0%	48.0%	36.9%	29.0%
Age at treatment initiation							
< 50	72	13.1 (9.8, 20.5)	**<0.001**	76.0%	55.6%	39.7%	34.7%
50 – 65	425	18.3 (12.6, 21.7)		76.6%	58.3%	50.5%	42.0%
66 – 75	851	13.0 (11.6, 14.6)		70.7%	52.8%	41.0%	32.6%
76+	764	11.9 (10.2, 12.6)		67.1%	48.7%	33.7%	25.5%
Treatment experience							
Naïve	1,994	12.9 (12.3, 14.1)	*	71.7%	53.4%	41.5%	33.2%
Experienced	118	7.2 (4.9, 9.1)		54.8%	39.0%	24.1%	18.5%
Drug							
Nintedanib	1,591	13.5 (12.6, 14.7)	**< 0.001**	74.2%	55.5%	41.9%	32.8%
Pirfenidone	521	8.4 (7.2, 10.3)		60.0%	43.2%	35.4%	29.5%
Diagnosis							
IPF	1,724	12.8 (12.0, 14.0)	0.948	70.7%	53.0%	40.9%	32.3%
PPF	388	12.0 (11.0, 13.1)		70.6%	50.0%	37.3%	31.7%

Note: [a] One naïve patient was excluded from this analysis as an outlier as he/she had 40 alternated prescriptions of Nintedanib and Pirfenidone during the follow-up.
*Not provided due to small power. Abbreviation: CI: confidence interval.

Table S7. Treatment persistence rates with death treated as a censoring event, under the base-case and gap-based (90-day) definitions, overall and stratified by sex, age at treatment initiation, and diagnosis.

**Treatment persistence in months**	**Base Case % persistent at**				**Sensitivity Analysis % persistent at**			
	**6 months**	**12 months**	**18 months**	**24 months**	**6 months**	**12 months**	**18 months**	**24 months**
**Overall**	94.6%	91.5%	89.0%	88.2%	70.7%	52.3%	40.2%	31.9%
Sex								
Male	94.5%	91.1%	88.8%	87.5%	72.1%	54.5%	41.8%	33.4%
Female	94.7%	92.2%	89.4%	89.4%	68.0%	48.0%	36.9%	29.0%
Age at treatment initiation								
< 50	98.2%	99.0%	99.0%	99.0%	76.0%	55.6%	39.7%	34.7%
50 – 65	97.1%	94.8%	93.5%	92.2%	76.4%	57.8%	50.1%	41.6%
66 – 75	94.7%	90.1%	86.6%	85.8%	70.7%	52.6%	40.9%	32.5%
76+	92.6%	90.6%	88.4%	87.7%	67.0%	48.5%	33.5%	25.1%
Diagnosis								
IPF	93.8%	90.2%	87.3%	86.2%	70.7%	52.7%	40.7%	32.0%
PPF	97.9%	97.5%	97.5%	97.5%	70.4%	49.9%	37.2%	31.6%

Note: [1]Log-rank test. Abbreviations: CI: confidence interval; IPF: idiopathic pulmonary fibrosis; MPR: medication possession ratio; NR: not reached; PPF: progressive pulmonary fibrosis; Q1-Q3: 1st-3rd quartile; SD: standard deviation.

Table S8. Observable reasons for treatment cessation based on prescription patterns.

Outcome during follow-up	Base-case definition	Gap-based definition (90 days)
Remained on treatment at the end of follow-up	1,541	905
Switched to alternative anti-fibrotic	182	168
Prolonged interruption with re-initiation of the same drug	—	359
Prolonged interruption without subsequent observable treatment re-initiation	—	516
Death	389	332
Total	2,112	2,112

Note: Treatment outcomes were inferred from prescription refill patterns. Reasons for treatment discontinuation (e.g., adverse events) were not available in the data source.

Table S9. Supporting information in variables selection for multivariable survival models and multivariable fractional regression models (Base case analysis). Results from univariate models regarding comorbidities.

N=2,088	**Univariate Cox models**		**Univariate Fine and Gray competing risk regression**		**Fractional logistic regression**	
	**HR (95% CI)**	**p-value**	**SHR (95% CI)**	**p-value**	**OR (95% CI)**	**p-value**
**Comorbidities, yes vs. no**						
Gastrointestinal disorders	1.18 (0.91, 1.53)	0.221	1.94 (1.11, 3.40)	**0.021**	1.06 (0.88, 1.28)	0.555
Cardiovascular disorders	1.38 (1.12, 1.69)	**0.002**	0.95 (0.68, 1.32)	0.738	0.98 (0.85, 1.14)	0.828
Dyslipidemia	1.28 (1.05, 1.56)	**0.015**	1.70 (1.16, 2.50)	**0.007**	1.04 (0.90, 1.20)	0.604
Diabetes mellitus	1.25 (1.05, 1.48)	**0.010**	1.22 (0.90, 1.65)	0.193	0.98 (0.85, 1.12)	0.747
Hyperuricemia	1.34 (1.11, 1.61)	**0.002**	0.91 (0.63, 1.30)	0.590	0.93 (0.80, 1.09)	0.377
Osteoporosis	0.98 (0.81, 1.20)	0.865	1.00 (0.71, 1.40)	0.983	0.90 (0.77, 1.05)	0.192
Infections	1.00 (0.77, 1.29)	0.976	1.02 (0.65, 1.59)	0.946	0.79 (0.65, 0.97)	**0.023**
Rheumatological disorders	0.86 (0.65, 1.13)	0.271	0.61 (0.35, 1.06)	0.078	0.86 (0.69, 1.07)	0.168
Neurological disorders	1.03 (0.60, 1.74)	0.926	0.93 (0.35, 2.46)	0.880	0.69 (0.48, 0.99)	**0.043**
Psoriasis	0.66 (0.27, 1.60)	0.359	1.20 (0.38, 3.77)	0.760	1.15 (0.64, 2.05)	0.648
Psychiatric disorders	0.68 (0.22, 2.12)	0.508	2.37×10^{-19} (1.30×10^{-19}, 4.32×10^{-19})	**<0.001**	0.87 (0.38, 2.01)	0.746
Thromboembolic diseases	2.55 (1.06, 6.15)	**0.038**	3.88 (1.29, 11.6)	**0.016**	1.88 (0.98, 3.62)	0.059

Notes: Comorbidities with less than 20 cases (as psychiatric disorders and thromboembolic diseases) were not considered for inclusion in the multivariable models. Cardiovascular disorders were associated with dyslipidemia, diabetes mellitus and hyperuricemia, as such only cardiovascular disorders were

included in the multivariable generalized gamma AFT regression model. Similarly, gastrointestinal disorders were associated with dyslipidemia, and only gastrointestinal disorders were included in the multivariable Fine and Gray competing risk regression model. Abbreviations: CI: confidence interval; HR: hazard ratio; OR: odds ratio; SHR: Sub-distribution Hazard Ratios.

Table S10. Associations between patient characteristics and treatment persistence: results from accelerated failure time, and competing risks regression models (Sensitivity analysis).

	**Generalized gamma AFT regression**				**Fine and Gray competing risk regression**			
	**Univariate model N=2,112**		**Multivariable model N=2,088**		**Univariate model N=2,112**		**Multivariable model N=2,088**	
	**TR (95% CI)**	**p-value**	**TR (95% CI)**	**p-value**	**SHR (95% CI)**	**p-value**	**SHR (95% CI)**	**p-value**
Sex								
Female vs. Male	0.92 (0.81, 1.04)	0.190			1.45 (1.27, 1.66)	**<0.001**	1.42 (1.22, 1.67)	**<0.001**
Age at treatment initiation								
< 50 years vs. 50 – 65 years	0.97 (0.68, 1.39)	**<0.001**	0.91 (0.64, 1.29)	**0.004**	1.37 (0.997, 1.89)	0.134	1.29 (0.94, 1.79)	0.209
66 – 75 years vs. 50 – 65 years	0.71 (0.60, 0.84)		0.78 (0.66, 0.92)		1.10 (0.92, 1.31)		1.16 (0.97, 1.39)	
76+ years vs. 50 – 65 years	0.63 (0.54, 0.75)		0.72 (0.61, 0.86)		0.99 (0.82, 1.19)		1.06 (0.87, 1.28)	
Diagnosis								
PPF vs. IPF	1.17 (0.996, 1.38)	0.056	0.99 (0.83, 1.17)	0.861	1.17 (0.99, 1.37)	0.062	1.01 (0.85, 1.21)	0.879
Drug								
Pirfenidone vs. Nintedanib	0.54 (0.47, 0.62)	**<0.001**	0.56 (0.49,0.64)	**<0.001**	1.09 (0.93, 1.28)	0.305		
Comorbidities, yes vs. no								
Cardiovascular disorders	0.76 (0.66, 0.87)	**<0.001**	0.89 (0.76, 1.03)	0.104				
Gastrointestinal disorders	0.82 (0.68, 0.99)	**0.039**						
Dyslipidemia	0.83 (0.72, 0.96)	**0.012**						
Osteoporosis	0.84 (0.73, 0.97)	**0.014**			1.25 (1.08, 1.44)	**0.003**		
Hyperuricemia	0.84 (0.73, 0.97)	**0.014**	0.82 (0.71, 0.94)	**0.005**	1.25 (1.08, 1.44)	**0.003**	1.04 (0.89, 1.23)	0.607
Region (NUTS1)	**N=2,097**							
Aegean & Crete vs. Attica	0.93 (0.76, 1.15)	0.248			1.04 (0.84, 1.30)	0.187		
North Greece vs. Attica	0.94 (0.79.5, 1.10)				0.93 (0.78, 1.11)			
Central Greece vs. Attica	0.84 (0.71, 0.997)				1.12 (0.94, 1.34)			

Notes: p-value for "age at treatment initiation" was derived from an overall Wald test. Selection of comorbidities was based on the univariate models presented in **Table S6**. The event for AFT models was switch or death as combined event. The primary event for competing risk analysis was switch with death as competing risk. Variables with p<0.15 in the univariate level were entered in the multivariable models. Proportionality

of hazards was violated for treatment experience and drug. The generalised gamma AFT was selected, among other parametric survival models, based on AIC and BIC. In the generalised gamma acceleration AFT model, the coefficients are interpreted in terms of TRs rather than hazard ratios. The TR is derived as TR=eCoefficient, which represents the factor by which the time to the event changes per unit increase in the covariate. A TR <1 indicates shorter time to the event (higher risk), and a TR >1 indicates longer time to the event (lower risk). SHR focus on how covariates influence the probability of a specific event (e.g., switch) while accounting for the competing event (e.g., death). The SHR reflects the risk of the primary event in the presence of competing risks, not a combined outcome. A SHR >1 indicates an increased hazard (higher risk of the event), while a SHR <1 indicates a decreased hazard (lower risk of the event). Abbreviations: AFT: acceleration failure time; CI: confidence interval; IPF: idiopathic pulmonary fibrosis; NUTS1: Nomenclature of Territorial Units for Statistics level 1; PPF: progressive pulmonary fibrosis; SHR: Sub-distribution Hazard Ratios; TR: time ratio.

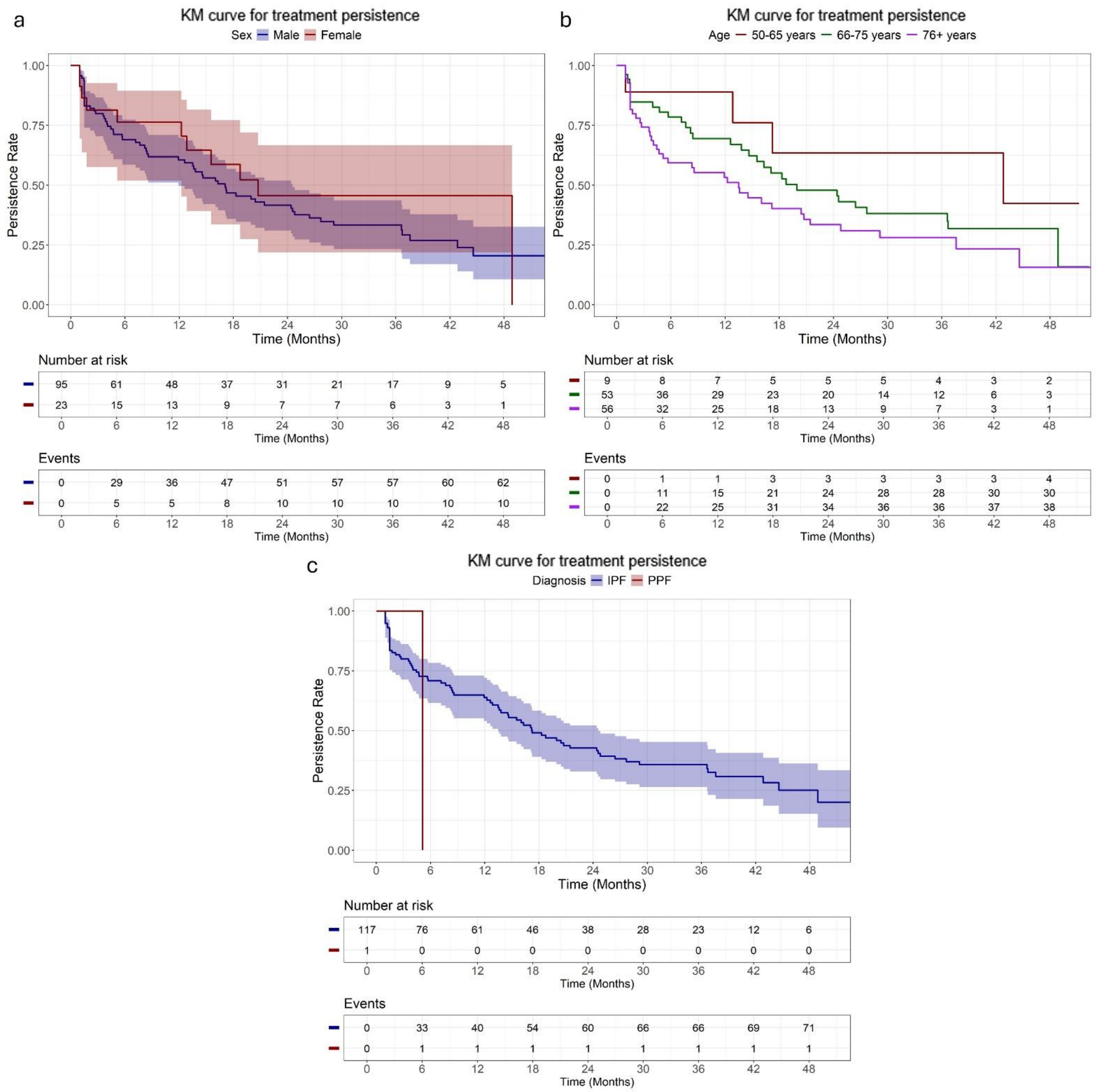

Figure S1. Kaplan - Meier curve of treatment persistence of experienced patients initiating anti-fibrotic treatment stratified by (**a**) sex, (**b**) age at treatment initiation, and c) diagnosis.
